# Capsaicin Exerts Antitumor Activity in Mesothelioma Cells

**DOI:** 10.3390/nu16213758

**Published:** 2024-11-01

**Authors:** Emanuela Andretta, Aurora Costa, Elisa Ventura, Massimiliano Quintiliani, Sara Damiano, Antonio Giordano, Andrea Morrione, Roberto Ciarcia

**Affiliations:** 1 Sbarro Institute for Cancer Research and Molecular Medicine, Center for Biotechnology, Department of Biology, College of Science and Technology, Temple University, Philadelphia, PA 19122, USA; emanuela.andretta@unina.it (E.A.); ac.auroracosta@gmail.com (A.C.); elisa.ventura83@gmail.com (E.V.); antonio.giordano@temple.edu (A.G.); 2 Department of Veterinary Medicine and Animal Productions, University of Naples Federico II, 80137 Naples, Italy; sara.damiano@unina.it (S.D.); rciarcia@unina.it (R.C.); 3 Department of Chemical Sciences, University of Naples Federico II, via Cinthia, 4, 80126 Naples, Italy; 4 Department of Medical Biotechnologies, University of Siena, 53100 Siena, Italy; 5 SHRO Italia ETS, Via Sestriere 17, 10026 Candiolo, Italy; massimilianoquintiliani@gmail.com

**Keywords:** mesothelioma, capsaicin, migration, proliferation, cell cycle, cisplatin resistance, natural drugs, cancer, AKT, ERK1/2

## Abstract

Background/Objectives: Mesothelioma is an aggressive cancer with limited treatment options. Mesothelioma therapy often involves a multimodal approach including surgery, radiotherapy and chemotherapy. However, the prognosis for patients remains poor. Difficult diagnosis, late symptoms when the tumor is in an advanced stage and the onset of chemotherapy resistance make mesothelioma difficult to treat. For this reason, it is essential to discover new pharmacological approaches. Capsaicin (CAPS) is the active compound of chili peppers. Based on CAPS’s anticancer properties on various tumor lines and its chemo-sensitizing action on resistant cells, in this study, we evaluated the effects of CAPS on mesothelioma cells to assess its potential use in mesothelioma therapy. Methods: To evaluate antiproliferative effects of CAPS, we performed MTS assays on various mesothelioma cells, representative of all major mesothelioma subtypes. Transwell migration and wound-healing assays were used to examine the effect of CAPS on mesothelioma cell migration. We also determined the effects of CAPS on oncogenic signaling pathways by assessing the levels of AKT and MAPK activation. Results: In this study, we show that CAPS significantly reduces proliferation of both parental and cisplatin-resistant mesothelioma cells. CAPS promotes S-phase cell cycle arrest and inhibits lateral motility and migration of mesothelioma cells. Accordingly, CAPS suppresses AKT and ERK1/2 activation in MSTO-211H and NCI-H2052 cells. Our results support an antitumor effect of CAPS on cisplatin-resistant mesothelioma cells, suggesting that it may reduce resistance to cisplatin. Conclusions: Our results could pave the way for further studies to evaluate the use of CAPS for mesothelioma treatment.

## 1. Introduction

Malignant mesothelioma (MM) is a rare and very aggressive tumor arising from the mesothelium, the thin membrane that lines several body cavities, such as pleura, peritoneum, pericardium, and tunica vaginalis testis [1]. Malignant pleural mesothelioma (MPM) is the most common in patients (73–85% of mesothelioma cases) [2] and approximately 3500 people are diagnosed with mesothelioma each year in the USA [3]. Mesothelioma predominantly affects men and women in a ratio of 5:1 [2]. Globally, approximately 29,000 mesothelioma deaths occurred in 2019 [4]. MM mostly occurs in patients older than 65 years, and its onset is in general associated with asbestos exposure, after a latency period of 20–50 years [2,5,6,7]. The International Agency for Research on Cancer (IARC) classified asbestos as a carcinogenic substance for humans (Group I) [8]. Several countries have banned the use of asbestos, but, unfortunately, it still represents a global health concern [9].

However, asbestos-unrelated mesothelioma has been diagnosed in young patients carrying germline mutations in BRCA1-associated protein 1 (BAP1) or other tumor suppressor genes [10,11].

MPM is associated with an estimated overall survival of 38% at 1 year and only 7% at 3 years from diagnosis [12]. According to histological features, MM is classified into three main subtypes: epithelioid, sarcomatoid and biphasic, which are associated with a different prognosis [13,14]. In fact, patients diagnosed with sarcomatoid MPM present a lower median survival (4 months) than those affected by epithelioid (19 months) or biphasic (12 months) subtypes [12]. The unfavorable prognosis of MPM patients is related to the late onset of symptoms compared to exposure to asbestos as well as a difficult diagnosis, often carried out when the tumor is in advanced stage [1,11,15,16,17].

The current options for MM therapy are limited. For pleural mesothelioma patients, the standard of care is cisplatin plus pemetrexed, and the combination of two immuno-checkpoint inhibitors, ipilimumab plus nivolumab [18,19]. Although these drug treatments improve patient survival, the prognosis of patients with MM remains poor [20,21]. MPM patients not candidates for surgery exhibited a 41.3% response rate to cisplatin plus pemetrexed in a phase III study [22]. However, the response rate to this combination treatment was significantly lower (26.3%) in other studies [23]. In addition, the development of resistance to chemotherapeutic drugs and the presence of mutations in *BAP1* gene result in a reduced response to cisplatin in MM patients [24]. Although the use of ipilimumab plus nivolumab has increased the overall survival of MPM patients compared to chemotherapy [25], the cost–benefit ratio makes ipilimumab plus nivolumab treatment a very limited approach [26,27,28]. Thus, there is the urgent need to develop new therapeutic approaches for treatment and interest in evaluating the beneficial properties of natural compounds in cancer [29,30,31,32].

Capsaicin (trans-8-methyl-*N*-vanillyl-6-nonenamide) (CAPS) is the main phytochemical compound responsible for the spicy flavor of red chili peppers. CAPS exhibits several beneficial properties on human health, including antibacterial, anti-inflammatory, analgesic, and anticancer activities [33]. In particular, CAPS exerts antiproliferative and antimigratory effects against various cancer cell lines [34,35,36,37,38]. It inhibits the activation of AKT and ERK1/2, serine-threonine kinases which are commonly involved in several cellular processes, including proliferation, cell growth, and migration [39,40,41,42,43]. In addition, CAPS promotes cancer cell apoptosis, and cell cycle arrest at different phases depending on cell lines and tumor models [39,44,45,46,47]. Interestingly, oral CAPS administration in xenograft mouse models of prostate cancer sensitized tumor cells to radiotherapy, resulting in a more marked reduction in tumor mass than radiotherapy alone [48,49]. Moreover, several studies have demonstrated a strong antitumor action of CAPS on cancer cells resistant to conventional chemotherapy. CAPS impairs cell cycle in various cisplatin-resistant cancer cell lines and, when combined with cisplatin, induces apoptosis in cisplatin-resistant gastric cancer cells [50,51]. However, the effects of CAPS on various MM cells have not been investigated. Given the properties of CAPS on other cancers, in this study, we evaluated the antitumor properties of CAPS in different MM lines, representative of major MM subtypes. Specifically, we show that CAPS exhibits strong antiproliferative action on both parental and cisplatin-resistant MM cells. Moreover, a further novel element of our study was to evaluate the effect of CAPS on migration and invasion, which are among the main hallmarks of tumor aggressiveness, in various mesothelioma lines. We provide the first evidence of the anti-migratory and anti-invasive action of CAPS and its inhibitory effect on AKT and ERK1/2 activation in MM cells. Our in vitro results are very encouraging and could pave the way for future clinical research on the use of CAPS as a possible therapeutic approach for patients with malignant pleural mesothelioma, even with the occurrence of cisplatin resistance.

## 2. Materials and Methods

### 2.1. Cell Lines and Reagents

MSTO-211H, NCI-H2052, NCI-H2452 and NCI-H28 human MM cell lines were purchased from ATCC (Manassas, VA, San Diego, CA, USA). These MM lines are representative of all MM subtypes: sarcomatoid (NCI-H2052), epithelioid (NCI-H2452, NCI-H28) and biphasic (MSTO-211H). All cell lines were cultured in RPMI-1640 medium containing GlutaMAX (Thermo Fisher Scientific, Waltham, MA, USA) supplemented with 1% penicillin–streptomycin (Pen/Strep) and 10% Fetal Bovine Serum (FBS) (R&D Systems, Minneapolis, MN, USA). The cells were kept at 37 °C in a humidified atmosphere containing 5% CO_2_.

### 2.2. Generation of Cisplatin-Resistant MM Cell Lines

Cisplatin-resistant MSTO-211H and NCI-H2052 cells were generated in our laboratory by long-term exposure to increasing concentrations of cisplatin (Calbiochem, San Diego, CA, USA) (5 µM, 10 µM, 15 µM, 20 µM, 25 µM), as described previously [52]. Following 72 h treatment with each concentration, the cells were grown in cisplatin-free medium for 10 days and then treated with the next higher concentration of cisplatin up to 25 µM. The cells were treated with cisplatin during their exponential growth phase. Selected resistant cells were maintained in RPMI-1640 medium containing 20 µM cisplatin (IC50) [53], 10% FBS and 1% Pen/Strep.

### 2.3. Capsaicin and Treatment Medium Preparation

Capsaicin (≥95%, from *Capsicum* sp.) (M2028) was purchased from Sigma Aldrich (St. Louis, MO, USA) and dissolved in Dimethyl sulfoxide (DMSO) to prepare a 200 mM stock solution. The stock solution was diluted in media to obtain the desired CAPS concentration used in the experiments.

### 2.4. MTS Assay

MSTO-211H, NCI-H2052, NCI-H2452 and NCI-H28 cells were seeded onto 96-well plates at a density of 1000 cells/well (MSTO-211H) or 2000 cells/well (NCI-H2052, NCI-H2452 and NCI-H28). The cells were treated for 24, 48 and 72 h with 5% FBS-RPMI 1640 medium containing CAPS at concentrations ranging from 50 µM to 200 µM for all MM cell lines, except for NCI-H28 cells, for which we used up to 300 µM.

Cell viability was assessed by the MTS assay (cat. no. G3582; CellTiter 96^®^AQueous One Solution Cell Proliferation Assay, Promega, Madison, WI, USA), following the manufacturer’s instructions. The absorbance at 490 nm was measured with a plate reader (Victor XS, Perkin Elmer, Waltham, MA, USA). The percentage of increase in cell growth from time zero (% increase over time 0) was calculated as follows:

% increase over time 0 = ((ABS Time T − ABS Time 0)/ABS Time 0) × 100,

where ABS Time T represented absorbance measured after 24, 48, or 72 h of treatment, while ABS Time 0 was absorbance at time zero.

### 2.5. Half-Maximal Inhibitory Concentration (IC50) Evaluation

Following 24, 48 and 72 h of CAPS treatment (50, 100, 200, 250 and 300 µM), IC50 values were calculated for MSTO-211H, NCI-H2052, NCI-H2452 and NCI-H28 cells by GraphPad Prism 8 software through a dose–response curve, selecting log(inhibitor) vs. normalized response-variable slope.

### 2.6. Cytofluorimetric Analysis of MM Cell Cycle

MSTO-211H (2.5 × 10^5^ cells), NCI-H2052 (3 × 10^5^ cells), NCI-H2452 (3 × 10^5^ cells) and NCI-H28 (3 × 10^5^ cells) were seeded onto 100 mm plates the day before treatment was started. After treatment for 48 or 72 h with 5% serum-RPMI 1640 medium containing DMSO or CAPS, the cells were fixed in cold 70% ethanol and stained with propidium iodide following instructions of the propidium iodide flow cytometry kit (Abcam, Cambridge, UK), as described previously [54]. Cell cycle analyses were performed by FACS (BD LSR II 14-Color Flow Cytometer, Biosciences) at the Wistar Institute Cytofluorimetry Core Facility (Philadelphia, PA, USA).

### 2.7. Wound-Healing Assay

A confluent monolayer of NCI-H2052, NCI-H2452 and NCI-H28 cells, seeded onto 6-well plates, was wounded using a 200 μL micropipette tip. After washing with Dulbecco’s phosphate-buffered saline (DPBS) (Gibco, Thermo Fisher Scientific), the cells were treated with medium containing DMSO or CAPS at concentrations that did not affect cell proliferation. Images of the wounded area were acquired by a DMi1 (Leica, Wetzlar, Germany) microscope (4× objective) both at time 0 (immediately after scratching) and at 24, 48 and 72 h of treatment until wound closure. The images were examined by using Wound_healing_size_tool update, an ImageJ/Fiji^®^ plugin [55,56]. The wound closure rate was measured as follows:

% wound closure = ((Wound area time 0 − Wound area time T)/Wound area time 0) × 100),

where wound area time 0 was the wound area after scratching, and wound area time T represented the wound area at the end of each treatment time.

### 2.8. Transwell Migration Assay

After starvation in serum-free medium (SFM) for 24 h, MSTO-211H, NCI-H2052, and NCI-H28 cells were seeded at a density of 2 × 10^4^ cells in SFM in the upper chamber of a 6.5 mm Transwell with an 8.0µm pore size polyester membrane insert (Corning REF 3464, Corning, NY, USA). SFM (for NCI-H2052) or 5% serum-RPMI 1640 medium (for MSTO-211H and NCI-H28) was introduced in the lower chamber. DMSO or CAPS were added to both chambers of the Transwell. Migration was measured at 16 h for NCI-H2052, 24 h for NCI-H28 and 27.5 h for MSTO-211H. After removing cells on the upper surface of the membrane using a cotton swab, the cells on the lower surface of the filter were fixed in ice-cold methanol and stained with Coomassie Brilliant Blue, as described previously [57]. Images were acquired by an inverted microscope (DMi1, Leica, Wetzlar, Germany).

### 2.9. Transwell Invasion Assay

The invasive capacity of MM cells was assessed using Transwell inserts with an 8 µm pore size membrane coated with homogeneous layer of Matrigel (Corning, NY, USA), as previously reported [57]. Briefly, 2 × 10^4^ NCI-H2052 cells were seeded, and invasion was stopped after 24 h of treatment in SFM containing DMSO alone or CAPS at a concentration of 100 µM. Cells on the upper surface of the membrane were removed, while cells on the lower surface of the membrane were stained with Coomassie Brilliant Blue after fixation in ice-cold methanol. Then, images were acquired by an inverted microscope (DMi1, Leica, Wetzlar, Germany).

### 2.10. Western Blotting

Cells were lysed using RIPA buffer (Thermo Fisher Scientific) supplemented with halt protease and phosphatase inhibitor cocktail (Thermo Fisher Scientific). Protein concentration was measured by the BCA assay (Thermo Fisher Scientific). The following primary antibodies pAKT S473 (#4060), pan-AKT (#4691), pERK1/2 (#4370), ERK1/2 (#9102) from Cell Signaling Technology (Danvers, MA, USA) and GAPDH (sc-365062) (Santa Cruz Biotechnology, Dallas, TX, USA) were used at a 1:1000 dilution, while the secondary antibodies anti-rabbit IgG-HRP-linked (sc-2357) and m-IgGk BP-HRP antibody (sc-516102) from Santa Cruz Biotechnology were used at a dilution of 1:5000. Chemiluminescence images of immunoblots were obtained using Odyssey Fc (LI-COR, Lincoln, NE, USA).

## 3. Results

### 3.1. Capsaicin (CAPS) Reduces Cell Proliferation Both in Parental and Cisplatin-Resistant Mesothelioma Cells

**Figure 1:**
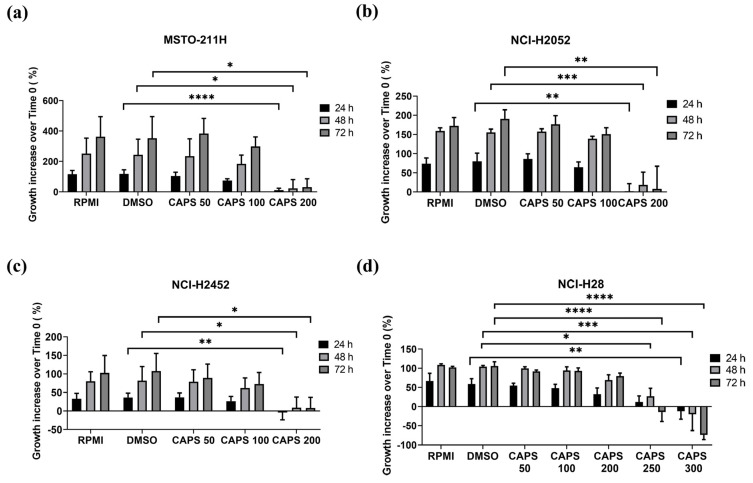
Capsaicin (CAPS) reduces proliferation of malignant mesothelioma (MM) cells. MSTO-211H (**a**), NCI-H2052 (**b**), NCI-H2452 (**c**), NCI-H28 (**d**) cells were treated with RPMI 1640 medium alone (RPMI); medium containing DMSO (DMSO) at the same %*v*/*v* of CAPS maximum concentration; and various CAPS concentrations (in µM) for 24, 48 and 72 h. Data are expressed as percentage of growth increase over time 0 and results are shown as mean ± standard deviation of three independent experiments. Statistical differences were evaluated by one-way analysis of variance (ANOVA) (* *p*-value < 0.05, ** *p*-value < 0.01; *** *p*-value < 0.001; **** *p*-value < 0.0001 versus DMSO).

To evaluate the anti-proliferative effect of CAPS on MM cells, we performed an MTS assay on several mesothelioma cell lines, representative of all MM subtypes, such as sarcomatoid (NCI-H2052), epithelioid (NCI-H2452, NCI-H28) and biphasic (MSTO-211H) (Figure 1). We assessed cell growth at 24, 48 and 72 h of treatment with various CAPS concentrations, and the results are expressed as % cell growth increase compared to time zero. To confirm that the effect of CAPS on cell proliferation was not related to cytotoxicity of DMSO used to dissolve CAPS, we also treated cells for 24, 48 and 72 h with 5% serum-RPMI 1640 medium containing DMSO at the same %*v*/*v* of cells treated with the highest concentration of CAPS. Cells treated in this way constituted the vehicle control (DMSO). As shown in Figure 1, no difference in cell growth was observed in vehicle control cells (DMSO) compared to cells exposed to RPMI 1640 medium (RPMI). Thus, no vehicle toxicity was detected since DMSO treatment did not affect cell growth of all MM lines tested.

Regarding CAPS treatments, concentrations of 50 µM and 100 µM did not impair growth of all MM cell lines when compared to DMSO alone. However, treatment with CAPS at 200 µM significantly reduced growth of MSTO-211H, NCI-H2052 and NCI-H2452 cells (Figure 1a, Figure 1b, Figure 1c, respectively), while it did not affect proliferation of NCI-H28 cells, which were resistant to these CAPS concentrations. Notably, CAPS concentrations at 250 µM and 300 µM significantly reduced growth of NCI-H28 cells (Figure 1d). Thus, CAPS shows strong anti-proliferative effect on MM cells when compared to DMSO-control-treated cells.

Then, we evaluated the IC50 following CAPS treatment for 24, 48 and 72 h for each MM line. The IC50 values are reported in Table 1.

As shown in Table 1, IC50 values were even lower for cells representing sarcomatoid (NCI-H2052) and biphasic (MSTO-211H) subtypes than for epithelioid (NCI-H2452 and NCI-H28) subtypes, suggesting the possible use of CAPS in particularly aggressive MM subtypes.

**Figure 2:**
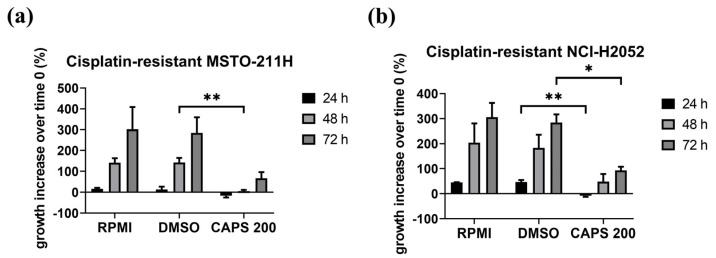
Capsaicin (CAPS) reduces proliferation of cisplatin-resistant malignant mesothelioma (MM) cell lines. Cisplatin-resistant MSTO-211H (**a**) and NCI-H2052 (**b**) cells were treated with RPMI 1640 medium alone (RPMI); medium containing DMSO (DMSO) at the same %*v*/*v* as CAPS concentration; medium containing CAPS at a concentration of 200 µM (CAPS 200) for 24, 48 and 72 h. Data are expressed as percentage of growth increase over time 0 and represent results obtained by two independent experiments performed in triplicate. Statistical differences were evaluated by one-way analysis of variance (ANOVA) (* *p*-value < 0.05, ** *p*-value < 0.01 versus DMSO).

Since occurrence of resistance to conventional chemotherapeutic drugs, such as cisplatin, results in treatment failure in patients [24,58,59], we evaluated the effect of CAPS on cell growth of cisplatin-resistant MSTO-211H and NCI-H2052 cells, generated in our laboratory following prolonged exposure to increasing concentrations of cisplatin [52], as described in Section 2. Significantly, treatment with CAPS 200 µM reduced cell proliferation of both cisplatin-resistant MSTO-211H and NCI-H2052 cells as compared to DMSO-treated cells (Figure 2a,b). Since no cytotoxicity of DMSO was observed in cisplatin-resistant cells, the antiproliferative effects were indeed related to CAPS action.

Collectively, we demonstrated the antiproliferative action of CAPS on all major MM subtypes. Furthermore, our results indicate that CAPS effectively reduces proliferation in cisplatin-resistant mesothelioma cells, suggesting that CAPS treatment could overcome cisplatin resistance of MM cells.

### 3.2. CAPS Impairs Cell Cycle in Mesothelioma Cells

**Figure 3:**
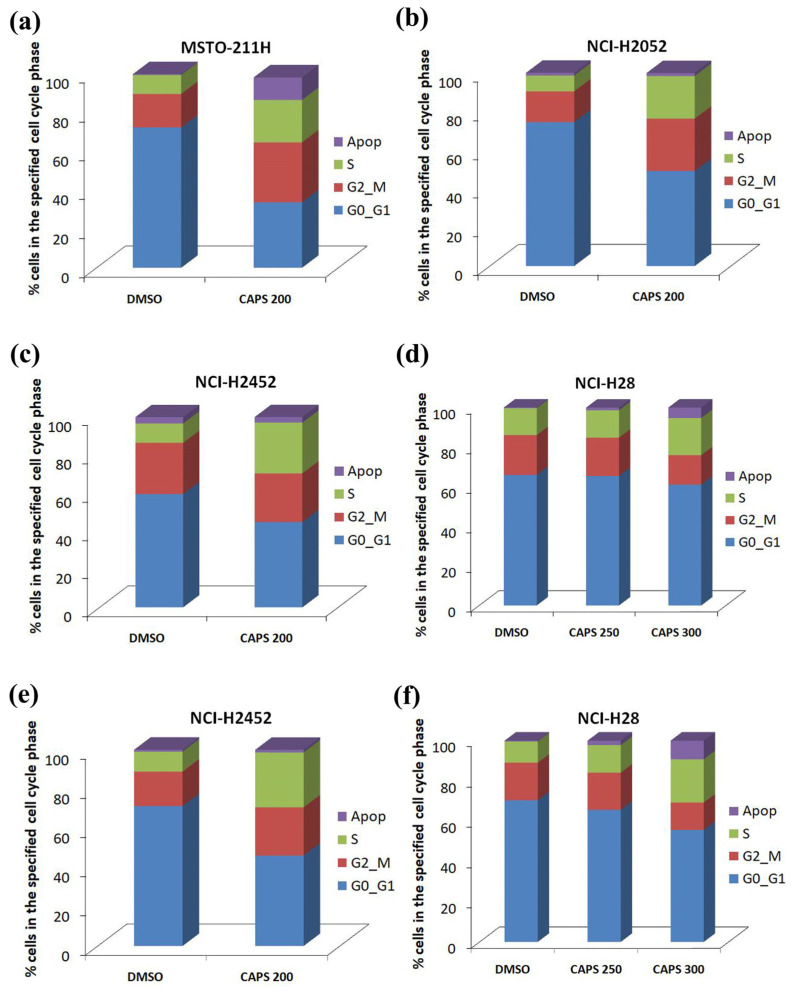
Capsaicin (CAPS) impairs cell cycle in malignant mesothelioma (MM) cells. Cell cycle analysis was performed in duplicate on MM cells after 48 h (**a**–**d**) or 72 h (**e**,**f**) treatment with medium containing Dimethyl sulfoxide (DMSO) used as vehicle at the same %*v*/*v* as the highest CAPS concentration or treatment with CAPS. The concentration of CAPS used for MSTO-211H (**a**), NCI-H2052 (**b**) and NCI-H2452 cells (**c**,**e**) was 200 µM (CAPS 200), while NCI-H28 cells (**d**,**f**) were treated with CAPS at the concentration of 250 μM (CAPS 250) and 300 μM (CAPS 300). Data show the impact of CAPS treatments on various cell cycle phases: G0-G1; S phase; G2-M and the percentage of apoptotic cells (Apop).

We then analyzed the effects of CAPS on the cell cycle and performed cytofluorometric analysis on MSTO-211H, NCI-H2052, NCI-H2452 and NCI-H28 cells following 48 h of CAPS treatment. As shown in Figure 3a, in MSTO-211H cells, CAPS reduced the fraction of cells in the G0/G1 phase (33.85% ± 8.27 in CAPS 200 versus 72.5% ± 3.54 in DMSO, * *p*-value < 0.05),while significantly increasing the S-phase cell population (21.9% ± 0.566 in CAPS 200 versus 9.8% ± 3.39 in DMSO, * *p*-value < 0.05). Furthermore, in MSTO-211H cells treated with CAPS 200 µM for 48 h, we observed an increase in the number of apoptotic cells compared to DMSO alone.

Consistent with the results obtained in MSTO-211H cells, CAPS promoted S-phase cell cycle arrest in NCI-H2052 cells (22.1% ± 3.68 in CAPS 200 versus 8.15% ± 1.91 in DMSO, * *p*-value < 0.05) (Figure 3b).

In NCI-H2452 cells, we detected an increase, although not significant, in the S-phase cell population following 48 h treatment with CAPS compared to vehicle-treated cells (Figure 3c).

Furthermore, 48 h treatment with CAPS at concentrations of 250 µM and 300 µM did not significantly impair the cell cycle of NCI-H28 cells. On the other hand, the number of apoptotic NCI-H28 cells was significantly increased following 48 h treatment with CAPS 300 µM (5.3% ± 2.69 in CAPS 300 versus 0.37% ± 0.55 in DMSO, * *p*-value < 0.05) (Figure 3d).

Since we did not observe significant differences in cell cycle distribution following treatment with CAPS for 48 h in both NCI-H2452 and NCI-H28 cells, we further investigated the effects of CAPS on cell cycle in these cell lines after 72 h of treatment (Figure 3e,f).

In NCI-H2452 cells, we detected a significant decrease in G0/G1-phase cell population following 72 h of treatment with CAPS at a concentration of 200 µM (46.10% ± 4.53 in CAPS-treated cells versus 71.3% ± 2.55 in DMSO-treated cells, * *p*-value < 0.05) (Figure 3e).

In contrast, in NCI-H28 cells, 72 h treatment with CAPS at a concentration of 250 µM did not impair cell cycle, while at a concentration of 300 µM, it resulted in significant cell accumulation in the S phase (21.45% ± 1.34 in CAPS 300-treated cells versus 10.57% ± 1.93 in DMSO-treated cells, * *p*-value < 0.05) (Figure 3f). In addition, the percentage of apoptotic NCI-H28 cells significantly increased in a dose-dependent manner following 72 h CAPS treatment (Figure 3f).

Overall, these results suggest that the effects of CAPS on the cell cycle may differ in cells derived from different mesothelioma subtypes. In sarcomatoid NCI-H2052 and biphasic MSTO-211H mesothelioma cells, CAPS significantly arrested cells at the S phase following 48 h of treatment. In contrast, in epithelioid NCI-H28 cells, a significant increase in S-phase cell population was achieved only after 72 h of treatment. An enhanced percentage of cells in S phase could indicate an S-phase cell cycle arrest promoted by CAPS in MM cells. Indeed, the results of cytofluorimetric cell cycle analysis in association with those obtained by the MTS assay suggest that CAPS exerts an antiproliferative action by arresting MM cells in the S phase of the cell cycle.

### 3.3. CAPS Reduces Migration of Various Mesothelioma Lines

Increased migratory capacity represents an important property of cancer cells [60,61]. Thus, to evaluate whether CAPS affected the migration of mesothelioma cells, we utilized two approaches: wound-healing assays to test lateral cell motility in monolayer growth, and Transwell migration assays. We used a CAPS concentration of 100 µM for MSTO-211H, NCI-H2052 and NCI-H2452 cells, and 200 µM for NCI-H28 cells, concentrations that did not inhibit MM cell growth to rule out the possibility that the effect of CAPS on lateral motility, which is assessed in monolayer, could be affected by CAPS action on cell growth.

#### 3.3.1. CAPS Impairs Lateral Motility of Various Mesothelioma Lines

**Figure 4:**
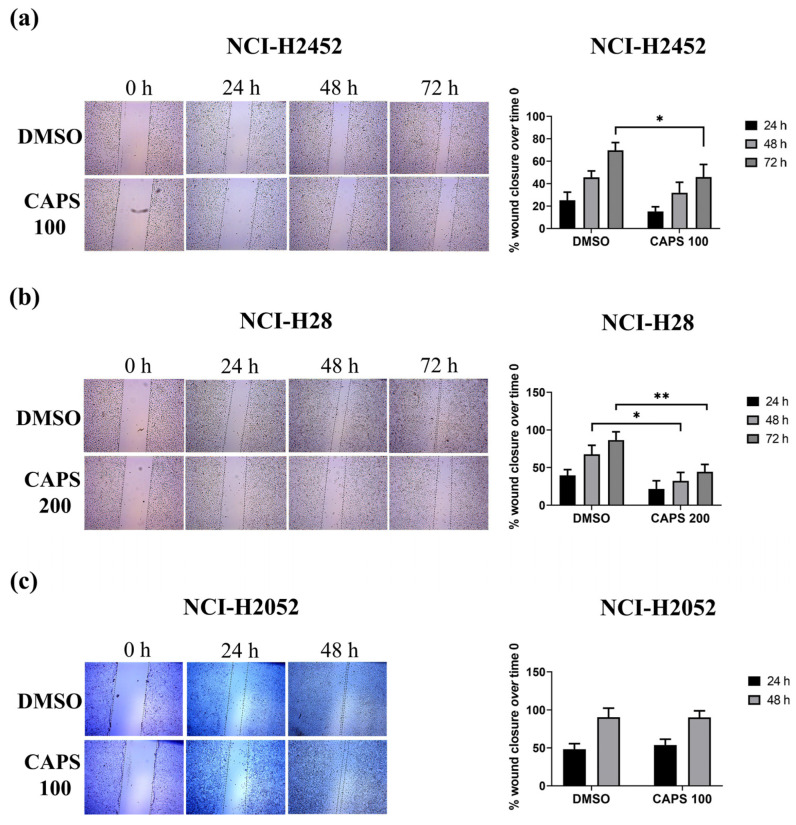
Capsaicin (CAPS) decreases lateral motility of NCI-H2452 and NCI-H28, but not of NCI-H2052 cells. Wound-healing assay was performed in NCI-H2452 (**a**), NCI-H28 (**b**) and NCI-H2052 (**c**) MM cells treated with medium containing either vehicle (DMSO) or CAPS at a concentration of 100 µM (CAPS 100) or 200 µM (CAPS 200) for 24, 48 and 72 h. Representative images of wound-healing assays in MM cells. Quantitative analysis of % wound closure. Data show the percentage of wound area closure compared with time 0. The results are expressed as mean ± standard deviation of three independent experiments. Statistical differences between DMSO and CAPS 100 µM or CAPS 200 µM were assessed by an unpaired *t*-test for each time of treatment (* *p*-value < 0.05 versus DMSO; ** *p*-value < 0.01 versus DMSO).

We conducted wound-healing assay on NCI-H2452, NCI-H28 and NCI-H2052 (Figure 4) cells, but not on MSTO-211H cells, as the latter tend to detach when they reach confluence.

In NCI-H2452 cells, 72 h of treatment with CAPS significantly reduced lateral motility compared to control cells treated with DMSO alone. Indeed, the mean value of % wound area closure was 69.62% in DMSO alone versus 45.90% in CAPS 100 µM after 72 h of treatment (* *p*-value < 0.05) (Figure 4a).

In NCI-H28 cells, treatment with CAPS 200 µM for 48 h significantly impaired lateral motility, as the percentage of wound area closure was 32.34% compared to 67.56% in control cells (* *p*-value < 0.05) (Figure 4b). In addition, the mean value of wound area closure percentage was 44.44% in cells treated with CAPS 200 µM for 72 h compared to 86.62% in the vehicle-treated control group (** *p*-value ≤ 0.001) (Figure 4b).

Unlike the other MM cell lines, lateral motility of NCI-H2052 was not affected by treatment with CAPS at a concentration of 100 µM, as shown in Figure 4c, suggesting that CAPS did not significantly decrease lateral motility of sarcomatoid-type cells.

Thus, CAPS significantly reduced lateral motility in the NCI-H28 and NCI-H2452 epithelioid MM lines, but not in NCI-H2052 cells, representative of the sarcomatoid type commonly associated with a more aggressive phenotype in patients. These results therefore indicate that the MM subtype could be important in modulating the response to CAPS and highlight the inhibitory action of CAPS on the lateral motility of epithelioid MM cells.

#### 3.3.2. CAPS Reduces Transwell Migration of Various Mesothelioma Cells

We then carried out a Transwell migration assay in NCI-H2052, MSTO-211H and NCI-H28 cells to evaluate the effect of CAPS on the anchorage-independent migratory ability of MM cells.

**Figure 5:**
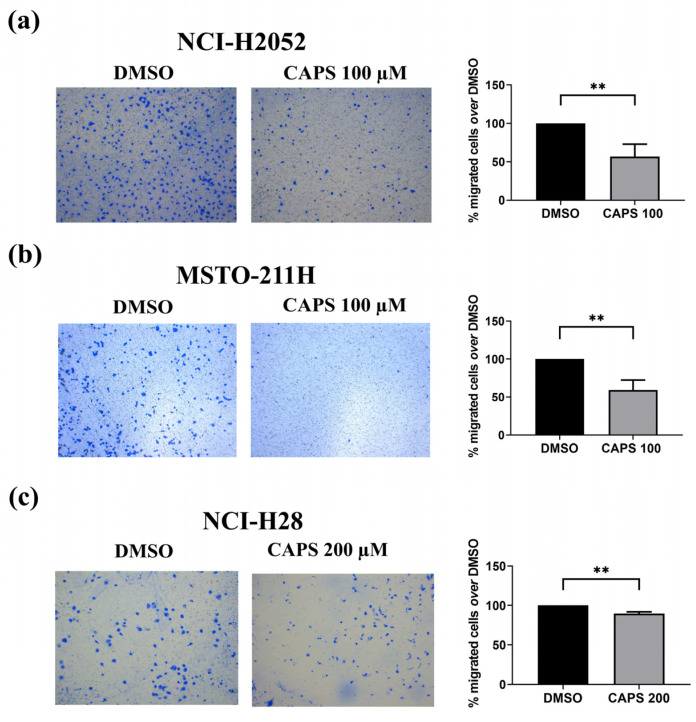
Capsaicin (CAPS) inhibits Transwell migration in malignant mesothelioma (MM) cells. Migration of NCI-H2052 (**a**), MSTO-211H (**b**) and NCI-H28 (**c**) cells, treated with medium containing DMSO alone (DMSO) or CAPS at a concentration of 100 µM (CAPS 100) or 200 µM (CAPS 200), was conducted by Transwell migration assays, as described in Materials and Methods. Representative images of MM cells. Quantitative analysis of percentage of migrated cells versus DMSO-treated cells. Data are expressed as mean ± standard deviation of three independent experiments. Statistical differences between DMSO and CAPS 100 µM or CAPS 200 µM were evaluated by an unpaired *t*-test (** *p*-value < 0.01 compared to DMSO).

CAPS exerted an antimigratory action on NCI-H2052 and MSTO-211H cells at a concentration of 100 µM, which did not affect cell proliferation. Indeed, CAPS treatment led to a reduction in the percentage of migrated NCI-H2052 and MSTO-211H cells compared to cells treated with medium containing DMSO alone (Figure 5a, Figure 5b, respectively).

For NCI-H28 cell migration, we used a CAPS concentration of 200 µM as this concentration did not affect the proliferation of this cell line. CAPS exposure at 200 µM significantly inhibited the migratory capacity of NCI-H28 cells as compared to DMSO-treated control cells (Figure 5c).

Collectively, these results demonstrate that CAPS inhibited the migration of MM cells, highlighting an additional important anticancer property of CAPS on MM cells. Thus, CAPS can reduce the aggressive phenotype of MM cells by suppressing the migration of MM cells.

### 3.4. CAPS Reduces Invasive Ability of Mesothelioma Cells

**Figure 6:**
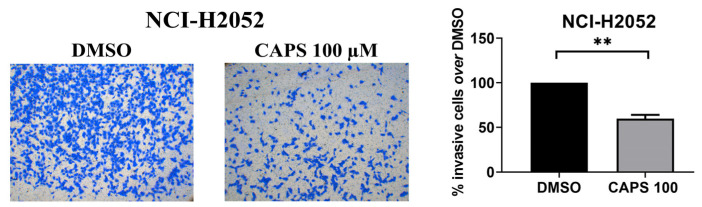
Capsaicin (CAPS) reduced the invasive capacity of NCI-H2052 cells. Invasion of NCI-H2052 cells, treated with medium containing DMSO alone (DMSO) or CAPS 100 µM (CAPS 100), was performed by Transwell invasion assays using Matrigel-coated chambers. Representative images of Transwell invasion assay on NCI-H052 cells. Quantitative analysis of percentage of invasive cells versus DMSO-treated cells. Statistical differences between DMSO and CAPS 100 µM were evaluated by an unpaired *t*-test (** *p*-value < 0.01 compared to DMSO).

The ability of cancer cells to migrate and invade through tissues is a feature underlying the process of tumor metastasis [62,63]. Thus, we evaluated the effect of CAPS on the invasion of mesothelioma cells. We focused on NCI-H2052 sarcomatoid-type mesothelioma cells since several studies have revealed the presence of distant metastases originating from sarcomatoid-type mesothelioma [64,65,66,67]. Thus, we treated NCI-H2052 mesothelioma cells with medium containing either the vehicle alone or CAPS at a concentration of 100 µM that did not impair cell growth, and we found a reduction in the ability of CAPS-treated NCI-H2052 MM cells to invade through Matrigel-coated Transwell chambers (Figure 6).

This result evidenced the anti-invasive action of CAPS in MM cells, specifically the sarcomatoid MM subtype. Given the highly aggressive nature and the ability of MM cells to metastasize, the anti-invasive action exerted by CAPS on sarcomatoid-type MM cells could represent a valid pharmacological approach for future studies.

### 3.5. CAPS Inhibits the Activation of the AKT and ERK1/2 Signaling Pathways in MM Cells

AKT and ERK1/2 are serine-threonine kinases with a critical role in the modulation of proliferation and migration of cancer cells [41,42].

**Figure 7:**
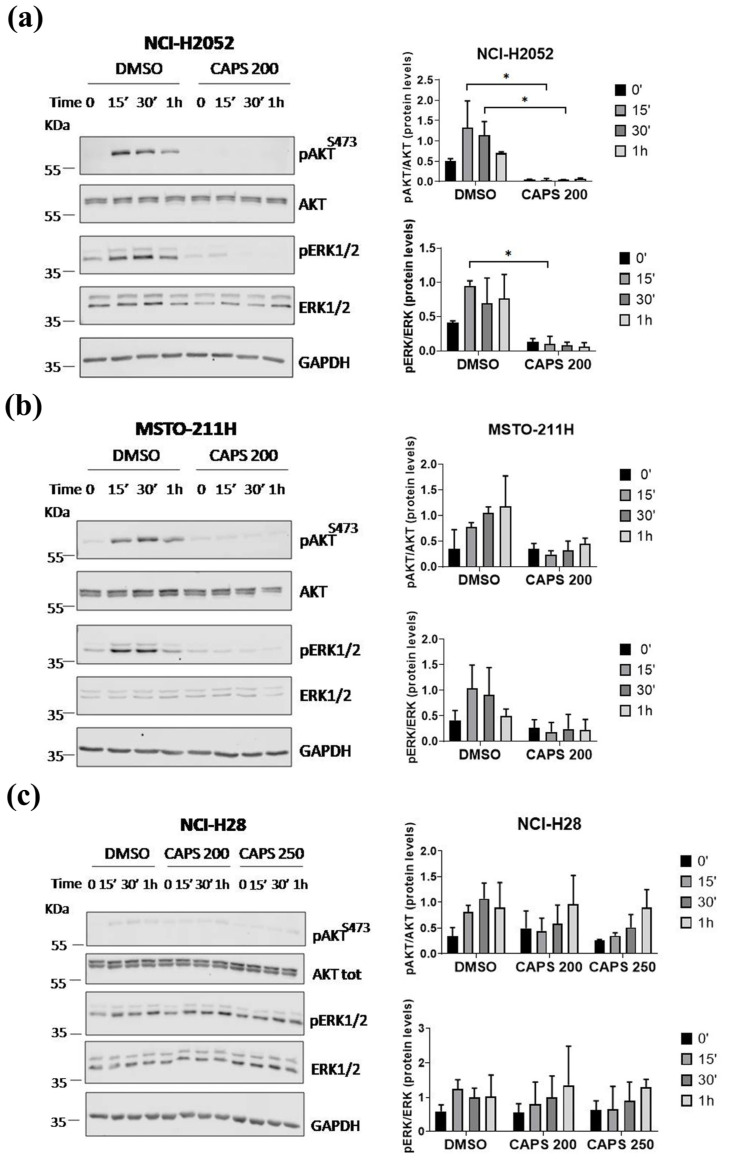
Capsaicin (CAPS) impairs AKT and ERK1/2 activation in malignant mesothelioma (MM) cells. Levels of total and phosphorylated AKT and ERK1/2 were evaluated by Western blotting in NCI-H2052 (**a**), MSTO-211H (**b**) and NCI-H28 (**c**) cells serum-starved for 5 h and then treated with 1% fetal bovine serum medium containing DMSO alone (DMSO) or CAPS at concentrations of 200 µM (CAPS 200) or 250 µM (CAPS 250) for 15′, 30′ and 1 h. Representative Western blotting images of two independent experiments (n = 2) for phosphoAKT (pAKT), total AKT (AKT), phosphoERK1/2 (pERK1/2), total ERK1/2 (ERK1/2) and GAPDH are shown. Densitometric analysis of phospho AKT/total AKT ratio and phospho ERK1/2/total ERK1/2 ratio. The values are expressed as mean ± standard deviation. Statistical differences were evaluated by one-way analysis of variance (ANOVA) (* *p*-value < 0.05).

Thus, to initially characterize the mechanisms of CAPS action in inhibiting the proliferation, cell cycle progression and motility of MM cells, we tested the level of AKT and ERK1/2 activation by immunoblot in NCI-H2052 (Figure 7a), MSTO-211H (Figure 7b) and NCI-H28 (Figure 7c) cells with and without CAPS treatment.

The cells were serum-starved in SFM for 5 h and then stimulated with 1% FBS-containing media supplemented with either DMSO as control or CAPS (200 µM or 250 µM) for 15 min, 30 min and 1 h.

As shown in Figure 7a,b, while serum induced strong and sustained activation of AKT and ERK1/2 in both NCI-H2052 and MSTO-211H DMSO-treated control MM cells, CAPS significantly inhibited the activation of both signaling pathways.

The results were slightly different in NCI-H28 cells. Notably, NCI-H28 cells showed rapid activation of AKT, which was not as strong as demonstrated in NCI-H2052 and MSTO-211H cells, and strong ERK1/2 activation. Significantly, treatment with CAPS at concentrations of 200 µM or 250 µM only resulted in delayed activation of AKT in NCI-H28 cells, which was detectable at 1 h of serum stimulation (Figure 7c). In contrast, CAPS at a concentration of 200 µM did not markedly reduce ERK1/2 activation, whereas at a concentration of 250 µM, it resulted in delayed ERK1/2 activation (Figure 7c).

Collectively, these results suggest that CAPS significantly inhibited the activation of AKT and ERK1/2 in MSTO-211H and NCI-H2052 MM cells. Conversely, in NCI-H28 cells, treatment with CAPS 250 µM only resulted in delayed AKT and ERK1/2 activation, suggesting that additional pathways targeted by CAPS can contribute to sustaining proliferation and motility of NCI-H28 MM cells.

## 4. Discussion

MM is a very aggressive cancer mostly associated with asbestos exposure. MM has poor prognosis despite the use of surgery and chemotherapy drugs. Therefore, developing and discovering new pharmacological therapeutic approaches is crucial to improve the therapeutic strategies for this disease [20,68].

Capsaicin (CAPS) is a capsaicinoid with several beneficial health effects [33]. In vivo and in vitro studies demonstrated antitumor activity of CAPS in various tumor models [44,69]. However, its effect on various MM cells was not previously established. Thus, this study aimed at the characterization of the anticancer properties of CAPS on MM cells. Specifically, we investigated the effect of CAPS on proliferation, migration and cell cycle in various MM lines, representative of MM subtypes.

Here, we demonstrated that (a) CAPS inhibits cell growth of MM cells; (b) CAPS induces S-phase cell cycle arrest in various MM cells; (c) CAPS reduces lateral motility, migration in Transwell and invasion of MM cells; and (d) CAPS delays or inhibits AKT and ERK1/2 activation in MM cells. Our results prove a strong antitumor action of CAPS on MM cells.

Specifically, we demonstrated the anti-proliferative action of CAPS on various MM cell lines at CAPS concentrations that are in line with those reported in the literature showing anticancer action against other cancer cells [44].

FDA-approved pharmacological treatment for pleural mesothelioma patients includes either the use of cisplatin plus pemetrexed or the combination nivolumab plus ipilimumab [19]. Unfortunately, resistance to conventional chemotherapy drugs, such as cisplatin, affects response to MM therapy [24,58,59]. Thus, in this study, we investigated whether CAPS had an effect on growth of cisplatin-resistant cells.

We demonstrated that CAPS significantly inhibited cell proliferation of cisplatin-resistant MSTO-211H and NCI-H2052 MM cells. Our in vitro results support scientific research towards new pharmacological strategies for MM since CAPS treatment could overcome cisplatin resistance of MM cells and have possible future implications in the clinical arena. Accordingly, other studies proved the antitumor action of CAPS on other cisplatin-resistant tumor cells. In this regard, Catanzaro et al. demonstrated that CAPS exerted a cytotoxic action and promoted cell cycle arrest in cisplatin-resistant ovarian and cervical squamous carcinoma cells. The action of CAPS on these cisplatin-resistant tumor cells was even stronger than on cisplatin-sensitive tumor cells [50]. Indeed, CAPS sensitizes various tumor cells to chemotherapeutics or radiotherapy. Indeed, CAPS in combination with conventional therapeutic approaches increases their efficacy, as observed by in vitro and in vivo studies on various tumor models [48,49]. CAPS sensitizes multidrug-resistant cells to doxorubicin [70]. This suggests the importance of studying CAPS in combination with other therapeutic approaches in MM therapy to improve the efficacy of conventional treatments, especially considering the occurrence of drug resistance. Chemotherapeutic agents are commonly associated with various adverse effects. There is a growing interest in discovering the beneficial properties, such as antitumor properties, of natural compounds in the scientific community [71]. Furthermore, natural compounds can exhibit an interesting ability to synergize, significantly improving the efficacy of currently available treatments [70,72]. Since we have proven the antitumor action of CAPS on cisplatin-resistant MM cells, future studies could evaluate the potential synergistic effects of CAPS in combination with other natural compounds in MM therapy.

Several studies have shown that the antiproliferative action of CAPS on various cancer cells is associated with cell cycle arrest at various phases depending on cell line [45,47,73]. Specifically, CAPS promotes G0/G1-phase cell cycle arrest in leukemia, osteosarcoma, colon cancer and bladder cancer cells [38,39,73], as well as G2/M-phase arrest in KB squamous cell carcinoma and MDA-MB-231 breast cancer cells [43,47].

Here, we investigated the effect of CAPS on MM cell cycle and demonstrated that CAPS induced S-phase cell cycle arrest in various MM cell lines. Our results are consistent with those reported by Chang et al. [45] and Yoon et al. [74], proving CAPS-promoted S-phase cell cycle arrest in MCF-7 and BT-20 human breast cancer cells. Similarly, CAPS analogues and derivatives are well known to cause S-phase arrest in various cancer lines [75,76].

In this work, we demonstrated for the first time that CAPS inhibited the migration of several MM cell lines at concentrations that did not affect cell growth. Indeed, CAPS exerted its anti-migratory action on NCI-H28, NCI-H2452 and MSTO-211H cells, consistently with other studies proving anti-migratory action of CAPS on several cancer cell lines [34,35,77,78,79].

Although CAPS did not affect lateral motility of NCI-H2052 cells, it inhibited Transwell migration in this cell line. In this regard, it is worth pointing out that wound-healing and Transwell migration assays measure two different types of cells motility [80]. While the first assay assesses collective migration of confluent cell monolayers characterized by cell–cell interactions, the second one enables the analysis of migration of single cells capable of crossing a membrane [81,82]. Furthermore, we prove that CAPS treatment reduces the invasive capacity of NCI-H2052 cells. This result highlights another interesting antitumor property of CAPS against MM cells. Indeed, the process of metastasis formation is related to the ability of cancer cells to migrate and invade tissues [62,63]. Distant metastasis formation has been found in patients with MM [64,83,84], and therefore the identification of new substances or drugs that can reduce the migratory and invasive ability is crucial. Our results on sarcomatoid MM cell invasion following CAPS treatment are in line with other studies that demonstrated the anti-invasive action of CAPS on renal and breast cancer cells [35,77]. Here, we provide the first evidence of the anti-invasive effect of CAPS on sarcomatoid-type MM cells. These results are very promising considering that the prognosis of patients with sarcomatoid-type MM is poor, with a lower median survival than patients with biphasic and epithelioid-type MM [14,85,86,87].

Given the antiproliferative and antimigratory properties of CAPS on different MM lines, we investigated the effect of CAPS on the activation of AKT and ERK1/2, serine-threonine kinases involved in various cellular mechanisms such as cell proliferation, survival and migration [41,42]. In detail, we proved that CAPS treatment significantly inhibited both AKT and ERK1/2 activation in MSTO-211H and NCI-H2052 MM cells. Thus, we speculate that CAPS exerts its anti-proliferative and anti-migratory action by inhibiting AKT and ERK1/2 signaling pathways, which we, along with other authors, demonstrated are important for MM motility [57,88,89,90]. Our results are in line with other studies proving that the anti-proliferative, anti-migrative, anti-invasive and pro-apoptotic action of CAPS is related to AKT and ERK pathways inactivation on various tumor lines [40,78,91,92]. The inactivation of these pathways has also been discovered in combinatorial treatments of CAPS with other anticancer drugs. Indeed, CAPS enhances the cytotoxicity of chemotherapeutics or compounds with well-known antitumor action, such as sorafenib and erlotinib, through mechanisms involving AKT inhibition [93,94] in various tumor cells. In addition, CAPS in combination with cisplatin suppresses transforming growth factor-β1-induced epithelial–mesenchymal transition (EMT) through the activation of claudin 1 and inhibition of the PI3K/AKT/mTOR pathway in squamous cell carcinoma of the tongue [95]. In contrast, ERK inactivation was associated with EMT inhibition in nasopharyngeal carcinoma cells co-treated with CAPS and cisplatin. In these cells, CAPS synergizes with cisplatin, exhibiting higher efficacy than treatment with cisplatin alone [92]. Taken together, these data support the evidence that CAPS can inhibit AKT and MAPK action in various cancer models, including MM. Conversely, low concentrations of CAPS can lead to AKT and ERK1/2 activation, as well as stimulate the invasion and migration of colorectal cancer cells [96,97,98]. Some natural compounds with a known anticancer action can exhibit different biological action depending on concentrations used, as, in fact, low CAPS concentrations can promote cancer cell proliferation [97,99]. Similarly, prolonged exposure to chemotherapeutic agents such as cisplatin or gemcitabine has been associated with increased tumor mass in cancer models [100]. However, the CAPS concentrations we used are within the range of concentrations with proven antitumor action in various cancer lines and therefore far from 0.1 to 10 µM CAPS range for which prolonged exposure promotes colorectal cancer cell proliferation and migration [44,97]. However, understanding which concentrations might be toxic or not beneficial requires further investigation in animal models. The evaluation of the effect of CAPS as a promoter or inhibitor of tumor cell growth in murine models is quite complex. Oral administration of CAPS at a concentration of 2.5 mg/Kg body weight was effective in inhibiting tumor formation in murine models of pancreatic cancer [101]. On the contrary, other studies proved a carcinogenic effect of CAPS used at different concentration [102]. Future studies are therefore required to further define the impact of low or high concentrations of CAPS, using various routes of administration, in murine models of mesothelioma.

In addition, our cytofluorimetric analysis revealed an enhanced number of apoptotic MSTO-211H and NCI-H28 cells following CAPS treatment. Since CAPS promotes apoptosis in different cancer cell lines through caspase-3 activation [36,103,104], we investigated the effect of CAPS on the levels of the total and cleaved form of caspase-3 in various MM lines and found that CAPS did not promote the cleavage of caspase-3 in MM cells but this preliminary evidence needs further verification. Our results are in line with Cömertpay et al., who found no significant differences in caspase-3 activation in CRL-5946 MM cells following treatment with CAPS or vehicle alone, proving that CAPS did not induce caspase-3-dependent cell death in these cells [105]. In this regard, other caspase-3-independent mechanisms may be involved in CAPS-induced apoptosis. Indeed, some studies performed on breast cancer cells detected increased intracellular calcium levels and subsequent translocation of apoptosis-inducing factor (AIF) from the mitochondria to the nucleus following CAPS treatment, thus also providing evidence for CAPS-induced caspase-independent apoptotic mechanisms [106,107]. However, the effect of CAPS on MM cells apoptosis remains to be fully elucidated.

Although further studies are necessary to investigate the mechanism underlying the antitumor action of CAPS on MM cells, our results demonstrate both antiproliferative and antimigratory properties of CAPS in various MM and its potential use in MM therapy.

CAPS inhibits the growth of cisplatin-resistant MM cells supporting the feasibility and possible clinical validity of combinatorial treatments with CAPS and current therapeutic regimens for MM therapy. Furthermore, developing anticancer drugs with fewer adverse effects is crucial for cancer patients. Chemotherapeutic agents, including cisplatin, are often associated with nausea, neuropathy and other adverse effects [108,109]. Natural products could represent a winning choice to counteract these negative effects. Topical CAPS administration exhibits beneficial effects in reducing episodes of nausea and vomiting (ClinicalTrials.govNCT04918069) [110], as well as neuropathic pain in patients undergoing chemotherapy treatments [111].

Despite its beneficial properties, CAPS is a substance that can cause irritation and burning of mucosa [112]. However, CAPS exhibits low bioavailability and half-life regardless of the route of administration [113]. For this reason, current studies are investigating possible strategies to deliver CAPS, such as liposomes and microemulsions [112,114]. To evaluate the potential use of CAPS in the clinics, it is necessary to consider the pharmacokinetics and metabolism of CAPS. Following oral administration, CAPS is mostly metabolized in the liver. In human liver microsomes, CAPS is converted into three major metabolites: 16-hydroxycapsaicin, 17-hydroxycapsaicin and 16,17-dehydrocapsaicin [113,115]. In contrast, following topical CAPS administration, vanillylamine and vanillic acid represent the main metabolic products in the skin [113]. A study conducted on volunteers proved that oral administration of a CAPS-rich extract via gel capsules reduced glucose levels [116]. Pharmacokinetic studies of CAPS were performed from blood of these volunteers. The highest observed CAPS concentration was 2.47 ng/mL at 45 min, but CAPS levels were detectable up to 90 min, suggesting a potentially rapid CAPS absorption [116]. In addition, another study demonstrated that oral administration of CAPS and isoflavone promoted hair growth in subjects with alopecia [117], but no pharmacokinetic parameter of CAPS was reported.

This limited number of studies on patients or volunteers highlights the use of CAPS as a promising approach. Notably, in this work, we proved the anti-proliferative, anti-migratory and anti-invasive properties of CAPS on various MM cell lines and its growth inhibitory action on cisplatin-resistant MM cells, thereby supporting a possible use of CAPS for MM therapy.

## 5. Conclusions

Mesothelioma is associated with poor prognosis. The development of new therapeutic strategies is an essential goal in the fight against mesothelioma. Considering the intriguing antitumor properties of CAPS in other tumor models, this work investigated the effect of CAPS on proliferation, migration and invasion of various MM cells, representative of MM subtypes. Specifically, CAPS inhibits the proliferation of both parental and cisplatin-resistant cells. These in vitro results strongly support the possible use of CAPS in clinical practice. However, further validation in 3D models or animal xenograft models is required before evaluating the use of CAPS in clinical therapy.

## Figures and Tables

**Table 1:** Capsaicin (CAPS) half-maximal inhibitory concentration (IC50) values on MM cells. IC50 values refer to CAPS concentrations in µM. IC50 was evaluated following 24, 48 and 72 h of CAPS treatment.

MM Cell Line	IC50 (24 h Treatment)	IC50 (48 h Treatment)	IC50 (72 h Treatment)
MSTO-211H	193.6	192.8	189.5
NCI-H2052	216.1	198.1	173.0
NCI-H2452	231.4	215.0	191.6
NCI-H28	325.2	275.9	242.4

## Data Availability

All data generated or analyzed during this study are included in this published article.
